# Repetitive negative thinking is associated with conflict-related neural activity that modulates evidence accumulation

**DOI:** 10.1016/j.xjmad.2026.100206

**Published:** 2026-09-20

**Authors:** Linisa Williams, Jessica Bomyea, Jonathon R. Howlett, Katia M. Harlé, Daniel M. Stout

**Affiliations:** a Department of Neurosciences, University of California San Diego, United States; b Department of Psychiatry, University of California San Diego, United States; c Center of Excellence for Stress and Mental Health, VA San Diego Healthcare System, United States

**Keywords:** Negative thinking, Conflict, Drift diffusion model, Inhibitory control, ERP

## Abstract

Repetitive negative thinking (RNT), including worry and rumination, is a transdiagnostic feature of anxiety and depressive disorders and is linked to altered cognitive control. However, the decision-level mechanisms underlying these alterations are not well understood. Here, we examined whether individual variation in RNT is associated with neural and computational markers of conflict processing. Thirty-seven participants completed a flanker task while undergoing electroencephalography, and inhibitory control dynamics were quantified using hierarchical drift diffusion modeling. Behaviorally, RNT was not associated with traditional behavioral markers of conflict processing. At the neural level, higher RNT was associated with a more negative midfrontal conflict N2. Computationally, higher RNT was associated with larger congruent-incongruent differences in drift rate. Similarly, more negative midfrontal N2 activity was associated with greater modulation of drift rate congruency effects. An exploratory mediation analysis indicated that the midfrontal conflict N2 significantly mediated the effect of RNT on drift rate differences between congruent and incongruent trials. These findings indicate that elevated RNT is associated with heightened conflict-related neural activity that increases the degree to which evidence accumulation is modulated by conflict, suggesting a more reactive and context-sensitive engagement of cognitive control.

## Highlights

• Repetitive negative thinking is associated with stronger midfrontal conflict N2.
• Repetitive negative thinking is linked with congruency effects on drift rate.
• Midfrontal conflict N2 is associated with congruency effects on drift rate.
• DDM drift rate and N2 may provide a neurocomputational account of negative thinking.

## 1 Introduction

Repetitive Negative Thinking (RNT) is a pervasive and transdiagnostic cognitive pattern characterized by persistent and recurrent thinking about personally relevant negative content, such as ruminating on past events or worrying about potential future threats [1]. RNT has been identified as a significant risk factor for the onset and persistence of depressive and anxiety disorders [2] and predicts poorer treatment outcomes from psychological interventions [3]. Although RNT is common across individuals, its more persistent and maladaptive forms may represent a dimensional feature of psychopathology, with increasing RNT associated with greater emotional distress and clinical impairment [4,5]. In addition to its impact on mental health, neurocognitive models propose that individuals with elevated RNT often become "stuck" in negative thought loops that compete with cognitive control functions required for goal directed behavior [6], particularly in detecting and resolving conflict [7]. This highlights the importance of understanding the underlying mechanisms through which RNT influences cognitive processes.

A central feature of cognitive control is the ability to detect and resolve conflict between competing sources of information [8,9]. When multiple pieces of information are presented at the same time, quick detection of the competing or conflicting information is required to be able to allocate cognition to what is most relevant to the task at hand. Converging neurocognitive models implicate the anterior cingulate cortex and medial frontal systems as key regions supporting conflict monitoring and control allocation [10]. Conflict-related processes can be measured using electrophysiological markers of medial frontal activity including the conflict N2, the error-related negativity, and midfrontal theta power [11,12]. Alterations in midfrontal EEG activity have been observed across neuropsychiatric disorders and reflect transdiagnostic markers of cognitive control dysfunction [13,14], with increased propensity to worry being associated with enhanced conflict-related N2 [15]. Disruptions in these conflict-related processes can result in reduced processing efficiency and impaired resolution of competition between choice alternatives, patterns consistently observed in individuals characterized by elevated RNT [16]. Importantly, variability in conflict-related behavior reflects multiple underlying latent neurocognitive mechanisms, rather than a single mechanism. Computational frameworks offer a method to dissociate these component processes, potentially providing mechanistic insight into how conflict is detected and resolved in individuals with elevated RNT [17].

One widely used framework for decomposing decision-making processes is the drift diffusion model (DDM) [18], which characterizes decisions as a process of noisy evidence accumulation in favor of one of two alternatives until a response threshold is met [19]. The DDM incorporates both reaction time and accuracy into a single model which disentangles distinct underlying latent mechanisms that may be differentially affected under conflict: drift rate (average rate of evidence accumulation toward the correct response) reflects the efficiency or speed and quality of information processing, boundary separation (distance between the upper and lower decision thresholds) measures response caution, reflecting the amount of evidence a participant requires before committing to a decision; and non-decision time (response time components outside of the decision process itself) captures perceptual encoding and motor processes. When paired with a cognitive control task, such as the Eriksen Flanker task [20], the DDM framework can reliably assess the specific effect of conflict on these separate component processes, for example is decision making inefficient, or is more evidence required when flankers are incongruent versus congruent [21]. At the neural level, distributed brain systems have been linked to latent components of decision-making within DDM frameworks [22], with midfrontal conflict-related activity corresponding to parameters governing evidence accumulation and response control [23]. At the clinical level, RNT is associated with disruptions in cognitive processing under conditions of conflict [24], including internally generated thought intrusions [25]. DDM approaches suggest that such disruptions may reflect alterations in latent decision-making components, including efficiency of evidence accumulation (drift rate), raising the question of whether RNT is associated with altered modulation of these processes under conflict beyond what is captured by mean reaction time and accuracy [26,27].

The current study aimed to examine the neurocomputational mechanisms underlying RNT during conflict processing. Here participants completed the well-validated Eriksen Flanker task [20] while EEG was recorded. We applied hierarchical DDM to test the hypothesis that RNT is associated with greater divergence in evidence accumulation between congruent and incongruent conditions, and to examine whether midfrontal conflict-related N2 activity is related to individual differences in evidence accumulation under conflict and to RNT.

## 2 Materials and methods

### 2.1 Participants

Forty-one adult participants were enrolled in the study. Four participants were excluded following data quality assessment (e.g., less than 50% of correct responses on either congruent or incongruent trials), resulting in a final analytic sample of 37 participants. Participants were recruited from the community with the goal of enrolling individuals spanning a broad range of RNT and internalizing symptoms. Recruitment methods included community advertising (e.g., flyers and advertisements on online platforms). Primary exclusion criteria during screening included: self-reported history of (1) bipolar disorder or psychotic symptoms, (2) severe alcohol or substance use disorder, (3) neurological conditions, and (4) current use of antipsychotic medications. Informed consent was obtained prior to participation, and participants were monetarily compensated for their time. The institutional review board of the University of California San Diego approved all study procedures.

### 2.2 Clinical and trait measures

#### 2.2.1 Repetitive negative thinking (RNT)

RNT was assessed using the Perseverative Thinking Questionnaire (PTQ; [28]). The PTQ is a 15-item self-report measure of transdiagnostic repetitive negative thinking (RNT) designed to assess non-specific intrusive, repetitive, and unwanted thinking. Participants rate each item on a 5-point Likert scale, with higher scores indicating greater RNT. Scores can range from 0 to 60.

#### 2.2.2 Generalized anxiety symptoms

Generalized anxiety symptoms were assessed using the Generalized Anxiety Disorder-7 scale (GAD-7 [29]). The GAD-7 is a 7-item self-report measure of anxiety symptoms over the past two weeks. Higher scores indicate greater generalized anxiety symptom severity. Scores can range from 0 to 21.

#### 2.2.3 Neuroticism

Trait neuroticism was assessed using the 12-item Neuroticism subscale from the Eysenck Personality Questionnaire-Revised Short Form (EPQ-RS [30]). This scale is a 12-item self-report measure of individual differences in emotional instability and propensity for negative emotionality. Scores can range from 0 to 12.

### 2.3 Flanker task

Participants completed a standard Eriksen flanker task [20] (see Fig. 1A). After a fixation cross (1200 – 1400 ms), participants were asked to quickly indicate the direction of the central arrow (left or right) that was presented for 200 ms. The central arrow was flanked by two arrows on each side that either matched (Congruent) the direction of the central arrow or mismatched (Incongruent); 50% congruency; condition type randomized across trials. Participants made button presses using their right hand. Participants completed 20 practice trials prior to completing 400 total trials (5 blocks: 80 trials each block; 200 trials per congruency). For analysis, trials with RTs ≤250 ms (2.76% of trials) were removed. All RTs were converted from milliseconds to seconds.

**Fig. 1:**
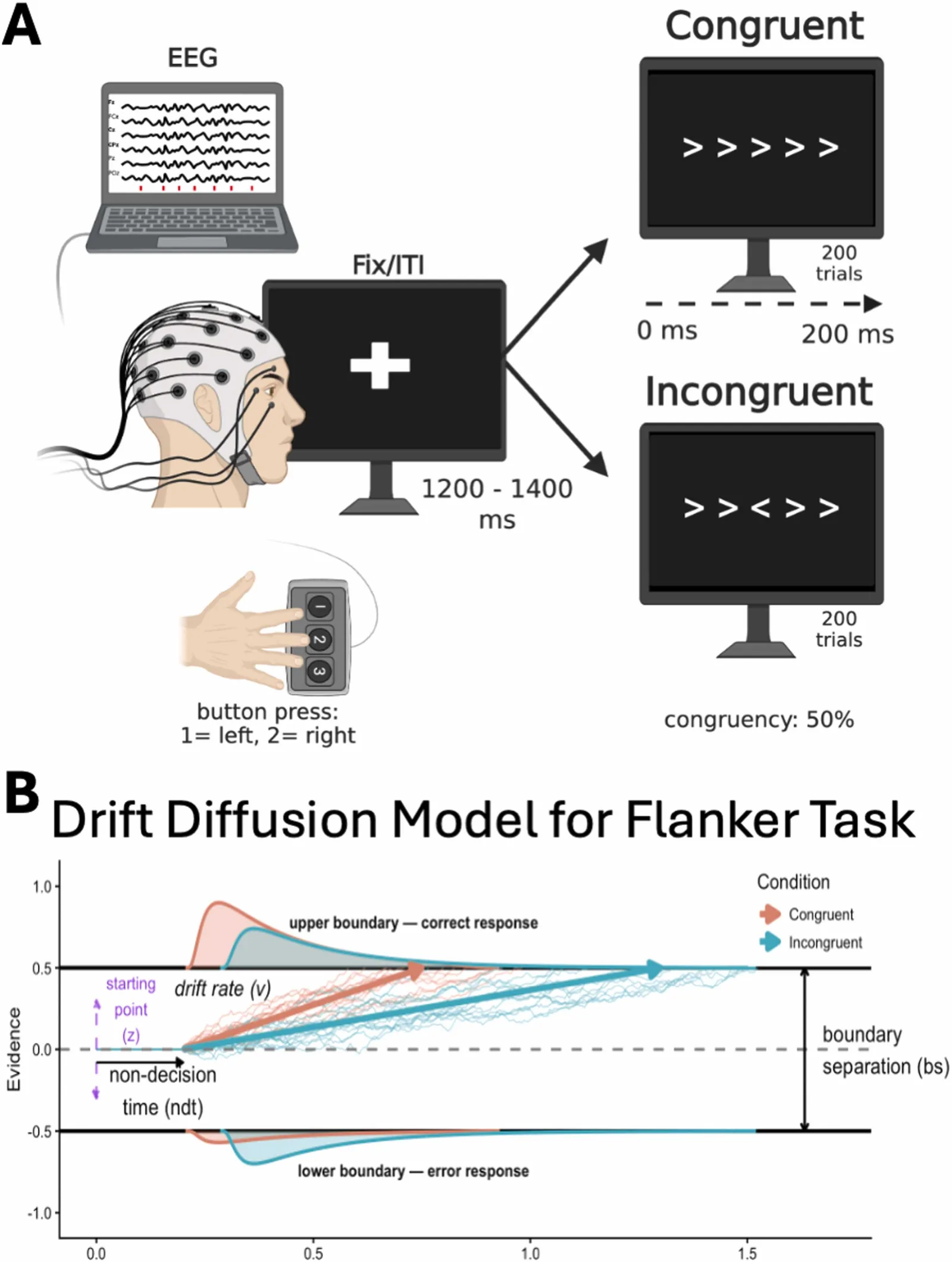
Flanker Task and Conceptual Application of DDM to the Flanker Task. (A) Participants completed an Eriksen Flanker task while electroencephalography (EEG) was recorded. See Methods Section 2.3 for details. Figure partially created using *BioRender.com*. (B) Conceptual drift diffusion model (DDM) applied to flanker task performance. Decisions are modeled as noisy accumulation toward correct (upper boundary) or incorrect (lower boundary) responses. Thin lines represent simulated trial-level evidence accumulation trajectories, while thicker arrows illustrate the average drift trajectories for congruent and incongruent trials. Here, congruent trials are depicted as faster, more efficient evidence accumulation, whereas incongruent trials show slower and less efficient accumulation due to response conflict. Key DDM parameters include drift rate (ν), boundary separation (*bs*), non-decision time (*ndt*), and starting point (*z*).

### 2.4 Hierarchical drift diffusion model

To examine how conflict influences latent decision-making processes in the flanker task, we fit a series of hierarchical drift diffusion models [31,32] using the brms package [32] using the *cmdstanr* backend within *R* [33], see Fig. 1B for a conceptual visualization. The models were hierarchical in that trial-level responses were nested within subjects, with parameters of interest estimated at both the group level and subject level. We estimated drift rate (ν), boundary separation (*bs*), and non-decision time (*ndt*). Bias (*z*) was fixed at 0.5 (0 in brms) since responses were coded as 1 (correct) or 0 (incorrect) [19]. The models were specified using a Wiener first-passage time distribution to jointly model reaction times (RTs, in seconds) and binary accuracy [34]. To characterize the effect of conflict, we specified a series of hierarchical drift diffusion models that varied in the extent to which core parameters were allowed to differ as a function of flanker congruency.

**Model 1**. Drift rate was modeled as a function of flanker congruency, with separate condition-specific group-level estimates and subject-level random slopes. Boundary separation and non-decision time were modeled with group-level intercepts and subject-level random intercepts. Relative starting-point bias was fixed at 0.5, corresponding to a fixed linear predictor of zero under the logit link used for the Wiener bias parameter, which assumes no systematic response bias. Model 1 tests whether flanker conflict effects are explained by changes in evidence accumulation alone.

**Model 2**. Model 2 was identical to Model 1, except that boundary separation was also specified to vary by flanker congruency, with separate condition-specific group-level estimates and subject-level random slopes. Non-decision time was modeled with group-level intercept and subject-level random intercepts. Bias was again fixed at 0.5 (0 in *brms*). Model 2 tests whether flanker conflict additionally reflects changes in response caution/decision-policy.

**Model 3.** Model 3 extended Model 2 by also modelling non-decision time as a function of congruency, where all 3 parameters were estimated with separate condition-specific group-level estimates and subject-level random slopes. Bias was again fixed at 0.5 (0 in *brms*). Model 3 tests whether flanker conflict extends to non-decision processes such as stimulus encoding or motor execution.

For each model, flanker congruency effects were parameterized using separate estimates for congruent and incongruent conditions rather than using a reference coding approach. For parameters that varied by congruency, subject-level effects were modeled as condition-specific random slopes, allowing subject-specific deviations to be estimated separately for each condition. Parameters that did not vary by congruency were modeled with subject-level random intercepts (see Supplemental Table 1 for summary of parameterization of each model). Correlations among random effects were not estimated to reduce model complexity and improve parameter identifiability and convergence. Weakly informative priors were used for all parameters, centered on plausible values for DDM parameters, with log link functions applied to boundary separation and non-decision time, and a logit link applied to bias to enforce parameter constraints (i.e., *bs* > 0, *ndt* > 0, 0 < *z* < 1). Models were estimated using four Markov chain Monte Carlo chains with 4000 iterations each (2000 warm-up). Informed initial values were used to ensure stable estimation and to satisfy parameter constraints (e.g., non-decision time < minimum RT). Model convergence was assessed using Gelman-Rubin $\hat{R}$ < 1.01 [35]. Posterior predictive checks evaluated the extent to which simulated data reproduced observed RT and summary statistics across conditions.

**Model comparison and fit.** The model selection strategy allowed for a principled evaluation of whether conflict-related effects are best characterized as arising from changes in evidence accumulation alone or whether they additionally reflect shifts in decision thresholds (response caution) and non-decision processes (encoding/motor). Model comparison was performed using Pareto-smoothed importance sampling leave-one-out cross-validation (PSIS-LOO-CV) via R::*loo* [36]. Relative model fit was evaluated using differences in expected log predictive density (ELPD) [37], with higher values indicating better out-of-sample predictive performance.

### 2.5 EEG recording and preprocessing

Continuous EEG was recorded using a 64-channel active electrode cap (ActiCap Slim Cap /ActiCHamp amplifier; Brain Products, Munch, Germany). Data were online referenced to the left mastoid. Impedances were kept below 25 kΩ. EEG was sampled at 1000 Hz. The vertical electrooculogram (VEOG) was measured using a pair of bipolar-referenced electrodes placed above and below the right eye. The horizontal electrooculogram (HEOG) was recorded using a pair of bipolar-referenced electrodes placed on the outer canthi of both eyes.

EEG data were processed using Brain Vision Analyzer (Brain Products GmbH, Germany; Version 2.0). Data were first filtered between 0.01 and 30 Hz via zero-phase shift band-pass (IIR Butterworth) filters. Bad channels were interpolated by spherical splines (*Mean* = 2.80, *SD*=2.09). Data were subsequently re-referenced offline to the average of the left and right mastoids. EEG data with ocular artifacts, including blinks and horizontal movements, were corrected using ocular independent components analysis [38], where the variance of each ICA component is calculated based on VEOG/HEOG channel activation and components with high correspondence to ocular activity were removed from the data. Data were segmented (-200–600 ms from arrow onset) and baseline corrected (-200 ms). Artifact rejection on trials was completed using automatic inspection within the time-window with a voltage step of 50 *µ*V/ms and a voltage difference of 100 *µ*V within a trial. On average, 1.99% (*SD*=3.76) of congruent, and 2.11% (*SD*=3.75) of incongruent trials were rejected due to artifact, *t* = -0.43*, p* = 0.67. Only correct responses were used for ERP specific analyses, with an average of 186.44 (*SD*=11.33) clean congruent trials and 160.93 (*SD*=36.96) clean incongruent trials retained for analysis.

In this study, we focused on the conflict-related N2 component elicited during the flanker task [11,39]. The N2 is a midfrontal negative-going ERP component. Here, the N2 was quantified as the difference between incongruent and congruent trials using pooled mean amplitude in the 280–400 ms window at midline frontal sites FCz and Cz. For four subjects, only Cz could be used due to amplifier malfunction for the electrode bundle containing FCz. The incongruent-minus-congruent N2 difference score reflects the degree of conflict-related processing, with more negative values indicating greater neural sensitivity to conflict.

### 2.6 Analytic strategy

Accuracy, reaction time, and N2 ERP analyses were conducted using paired samples *t*-tests to examine flanker congruency effects. From the best fitting DDM model, partially pooled posterior means for drift rate (ν) and non-decision time (*ndt*) were extracted per subject for each congruency condition, combining group-level (fixed) and subject-specific random effects via the *coef()* function in *brms*. Within-subject congruency effect contrasts were computed for each set of parameters. To examine relationships between RNT, conflict N2, and DDM parameters, Pearson correlations were conducted. To assess the specificity of RNT-related effects, we 1) repeated the primary Pearson correlation analyses between DDM parameter estimates and self-reported clinical and trait measures using generalized anxiety (GAD-7) and trait neuroticism (EPQ-RS-N), and 2) conducted a hierarchical multiple regression analysis where for each outcome (i.e., drift-rate congruency effect, midfrontal N2), GAD-7 and EPQ-RS-N were entered in Step 1, followed by RNT in Step 2 to assess for the additional variance explained by RNT.

**Exploratory mediation analysis**. We computed an exploratory mediation model using the R *lavaan* package [40], to test whether the midfrontal conflict N2 (incongruent – congruent) mediates the relationship between RNT and the drift rate congruency effect (Δν). Indirect, direct, and total effects were estimated with 95% bootstrap confidence intervals (5000 resamples), with effects considered supported when the 95% bootstrap confidence interval did not include zero.

All analyses were conducted in R [41] and JASP Version 0.19.3 [42].

## 3 Results

### 3.1 Participant characteristics

The analyzed sample included 37 adults (20 women). Participants had a mean age of 25.47 years (*SD* = 9.88); range = 18–52 and a mean of 14.33 (*SD* = 1.86) years of education. Participants identified as Asian (40.54%), Black (2.70%), White (29.73%), Multiracial (16.22%), or Other/preferred not to report (10.81%), 24.32% Hispanic, 70.27% as Non-Hispanic, and 5.41% Other/preferred to not report ethnicity.

Participants exhibited variability in RNT, with a mean PTQ of 23.3 (*SD* = 12.33; range = 0–47). Mean GAD-7 was 10.51 (*SD* = 3.12; range = 7–19) and mean EPQ-RS was 3.76 (*SD* = 3.04; range = 0–12).

### 3.2 Behavior results

As expected, accuracy was significantly higher for congruent (*Mean* = 0.95, *SE*=.007; range = 0.84–1.0), than incongruent (*Mean* = 0.87, *SE*=.014; range = 0.64–0.98) flanker trials, *t*(36) = 7.54, *p* < .001 (See Fig. 2A). Similarly, reaction times were significantly slower on incongruent trials (*Mean* = 442.23 ms, *SE*=9.32; range = 340.4 ms – 580.2 ms) compared to congruent (*Mean* = 396.39 ms, *SE*=8.59; range = 311.6 ms – 528.9 ms), *t*(36) = -15.33, *p* < .001, see Fig. 2B).

**Fig. 2:**
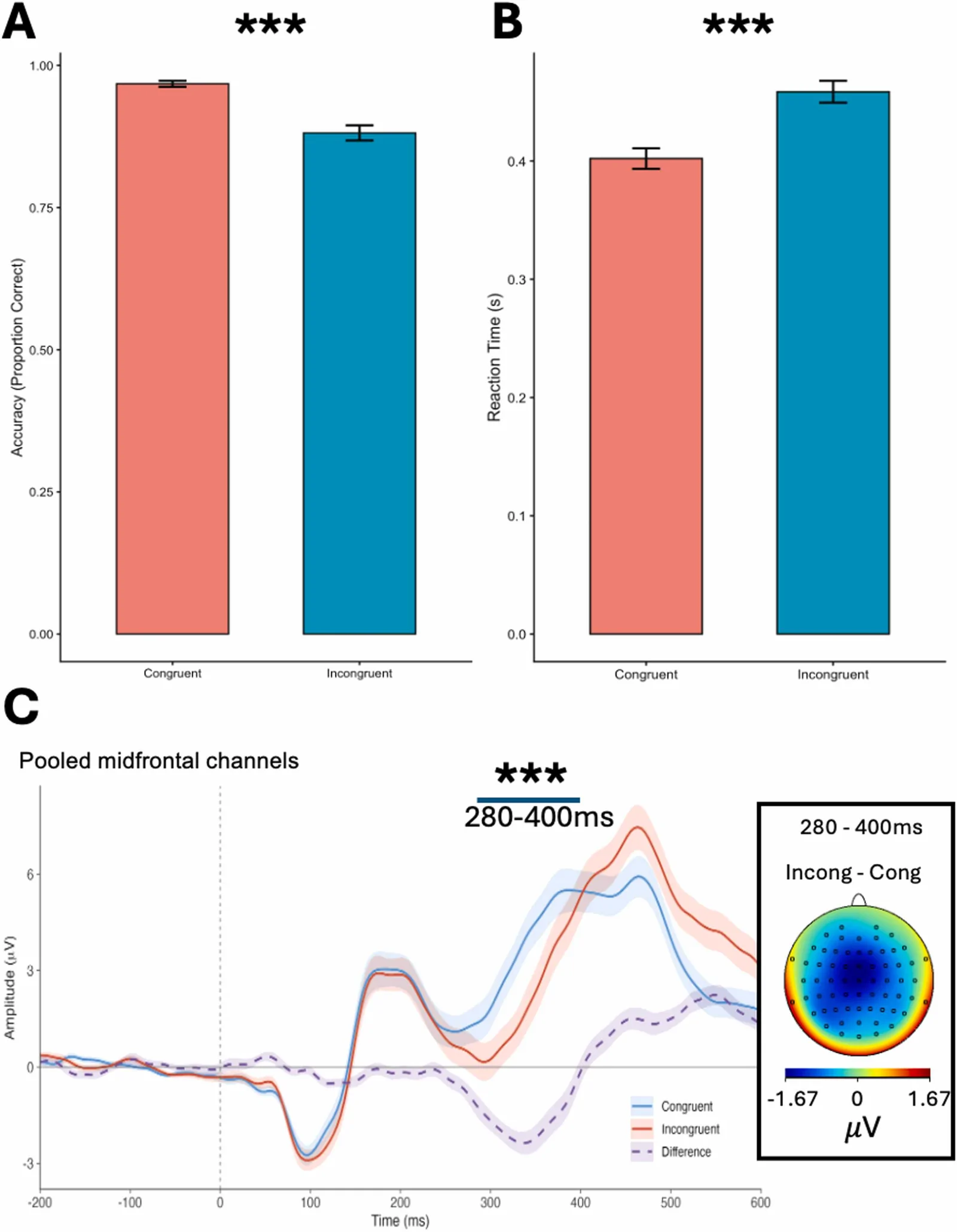
Behavioral and ERP results. Participants performed less (A) accurately and more (B) slowly on incongruent trials relative to congruent trials. (**C**) Conflict-related N2 was observed approximately 280–400 ms post-flanker onset. The N2 was significantly more negative for incongruent (red) than congruent (blue) trials. Dotted purple line depicts the incongruent minus congruent difference wave, with the expected negative peak. Scalp topography depicts the mean activity (μV) between 280 and 400 ms. Error ribbons depict standard error. ***p < .001.

### 3.3 ERP results

Incongruent trials (*Mean* = 2.77μV, *SE*=1.27) elicited a more negative-going (i.e., less positive) voltage than congruent trials (*Mean* = 4.43μV, *SE*=0.82) during the N2 window, *t*(36) = 5.86, *p* < .001, consistent with the expected conflict-related N2 effect (see Fig. 2C).

### 3.4 Computational model results

#### 3.4.1 Model fit

All models demonstrated good convergence across parameters ($\hat{R}$ < 1.01; see Supplemental Table 2). PSIS-LOO-CV indicated that Model 3 (congruency effects modeled for drift rate, boundary separation, and non-decision time) had the best fit relative to Model 2 (ΔELPD= −51.0, SE=21.3) and Model 1 (ΔELPD= −86.5, SE=38.2). Posterior predictive checks indicate Model 3 adequately reproduced the observed RT distributions for both congruent and incongruent trials and Markov chain convergence was confirmed via visual inspection of trace plots (see Supplemental Figures 1 and 2)**.** Parameters from Model 3 were used in subsequent analyses.

#### 3.4.2 Conflict effects on DDM parameters

Boundary separation was unexpectedly increased for congruent relative to incongruent trials (Δlog(bs)= 0.17, 95% CrI [0.04, 0.30]; Fig. 3-top row). As expected, drift rate was higher for congruent relative to incongruent trials (Δν was 1.65, 95% CrI [1.05, 2.24]; see Fig. 3-middle row). Non-decision time was slightly shorter for congruent trials (Δ*ndt= −12.19 ms,* 95% CrI [-19.06, −5.59]; Fig. 3-bottom row).

**Fig. 3:**
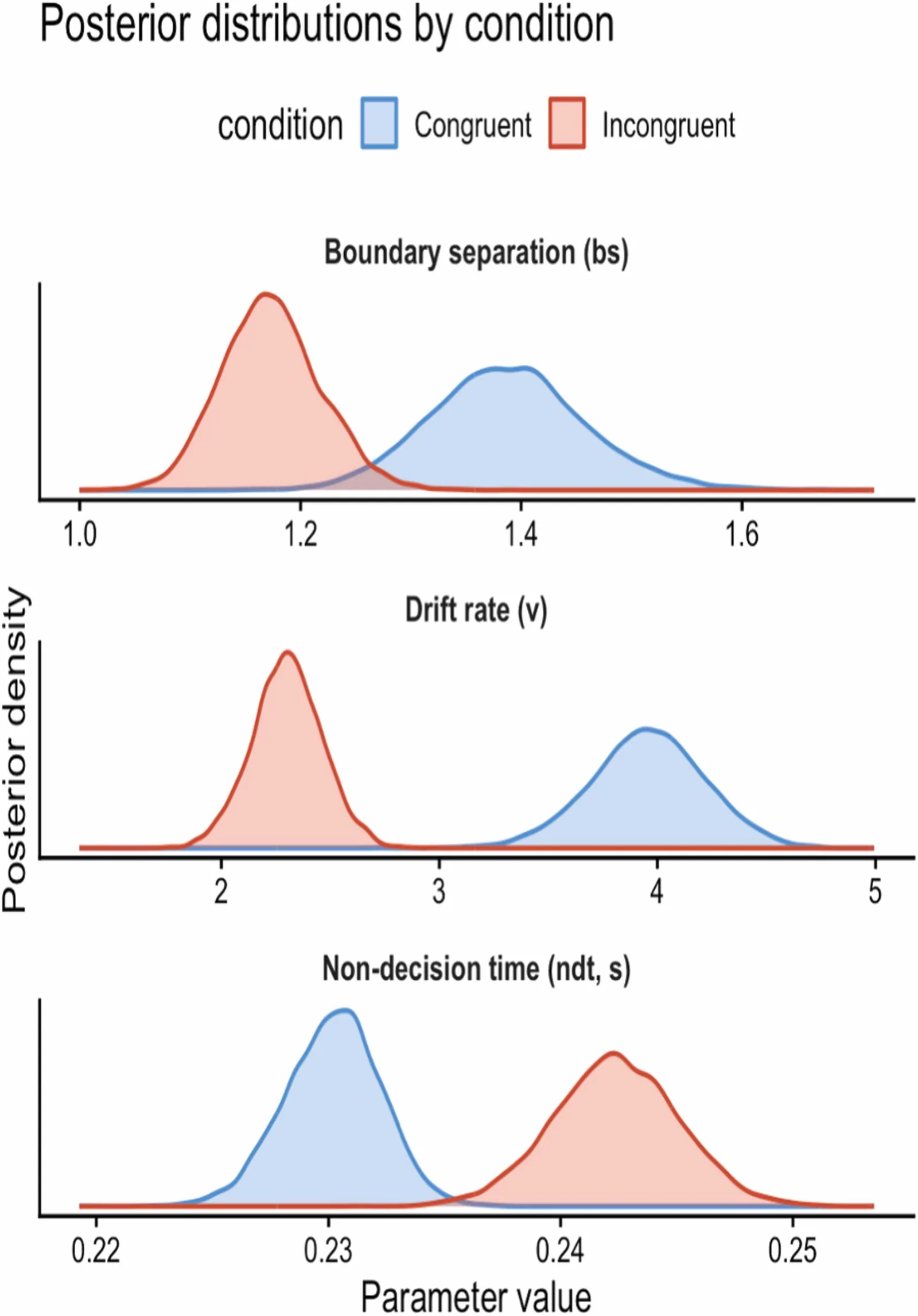
Posterior distributions of hierarchical drift-diffusion model parameters by condition. Posterior densities are shown for (top row) boundary separation (*bs*); (middle row) drift rates (ν), and (bottom row) non-decision time (*ndt*) for congruent (blue) and incongruent (red) flanker conditions.

Because of the unexpected direction of the boundary-separation effect, we conducted a post-hoc sensitivity analysis extending Model 3 to additionally account for across-trial variability in starting point (*s*_*z*_) [43,44]. The congruence effect on boundary separation persisted (Δlog(bs)= 0.14, 95% CrI [0.005, 0.272]), indicating that starting-point variability alone did not account for the effect. Given the unexpected direction the of the boundary separation effect and its persistence after accounting for starting point variability, we did not examine individual differences in boundary separation in relation to RNT or midfrontal N2 activity and focused instead on associations with drift rate and non-decision time (see Supplemental Materials and Supplemental Table 3).

#### 3.4.3 Relationship between RNT, N2, and DDM parameters

RNT was positively associated with differences (congruent – incongruent) in drift rate (Δν; *r*(35) = 0.37, *p* = .025), but not with non-decision time (Δ*ndt; r*(35) = -0.11, *p* = .51). However, RNT was not associated with traditional measures of flanker congruency effect on mean % accuracy or RT (*rs*< 0.26, *ps* > 0.13). RNT was also associated with stronger midfrontal N2 conflict activity (i.e., more negative incongruent minus congruent difference score), *r*(35) = -0.33, *p* = 0.047. Generalized anxiety symptoms and trait neuroticism were not significantly associated with drift rate congruency effects or midfrontal N2 (all *r*s < 0.23, *ps* > 0.17). Follow-up hierarchical regression analyses examined whether RNT explained additional variance beyond GAD-7 and neuroticism on drift rate congruency effect and midfrontal N2. RNT explained an additional 8% of variance in the drift-rate congruency effect (Δ*R*^2^ = 0.08, *p* = 0.085) and an additional 11% of variance in midfrontal N2 conflict activity (Δ*R*^2^ = 0.11, *p* = 0.052), indicating that RNT associations were attenuated when accounting for variation in generalized anxiety symptoms and neuroticism.

Midfrontal N2 differences (incongruent – congruent) were negatively associated with the drift rate congruency effect (congruent – incongruent; Δν; *r*(35) = -0.43, *p* = .009), but not congruency effect for non-decision time (Δ*ndt;* r(35)=0.16, *p* = .35), see Supplemental Figure 3 for scatter plots.

**Exploratory mediation analysis.** The total effect of RNT on drift rate congruency effect was significant (total effect: Β = 0.035, 95% bootstrap CI [0.010, 0.057]). N2 conflict amplitude showed a significant indirect effect in the relationship between RNT and the drift-rate congruency effect (indirect effect: Β = 0.011, 95% bootstrap CI [0.001, 0.025]; standardized indirect effect = 0.11), see Fig. 4. After accounting for midfrontal N2, the direct effect of RNT on drift rate was not significant (Β = 0.024, 95% bootstrap CI [-0.004, 0.048]).

**Fig. 4:**
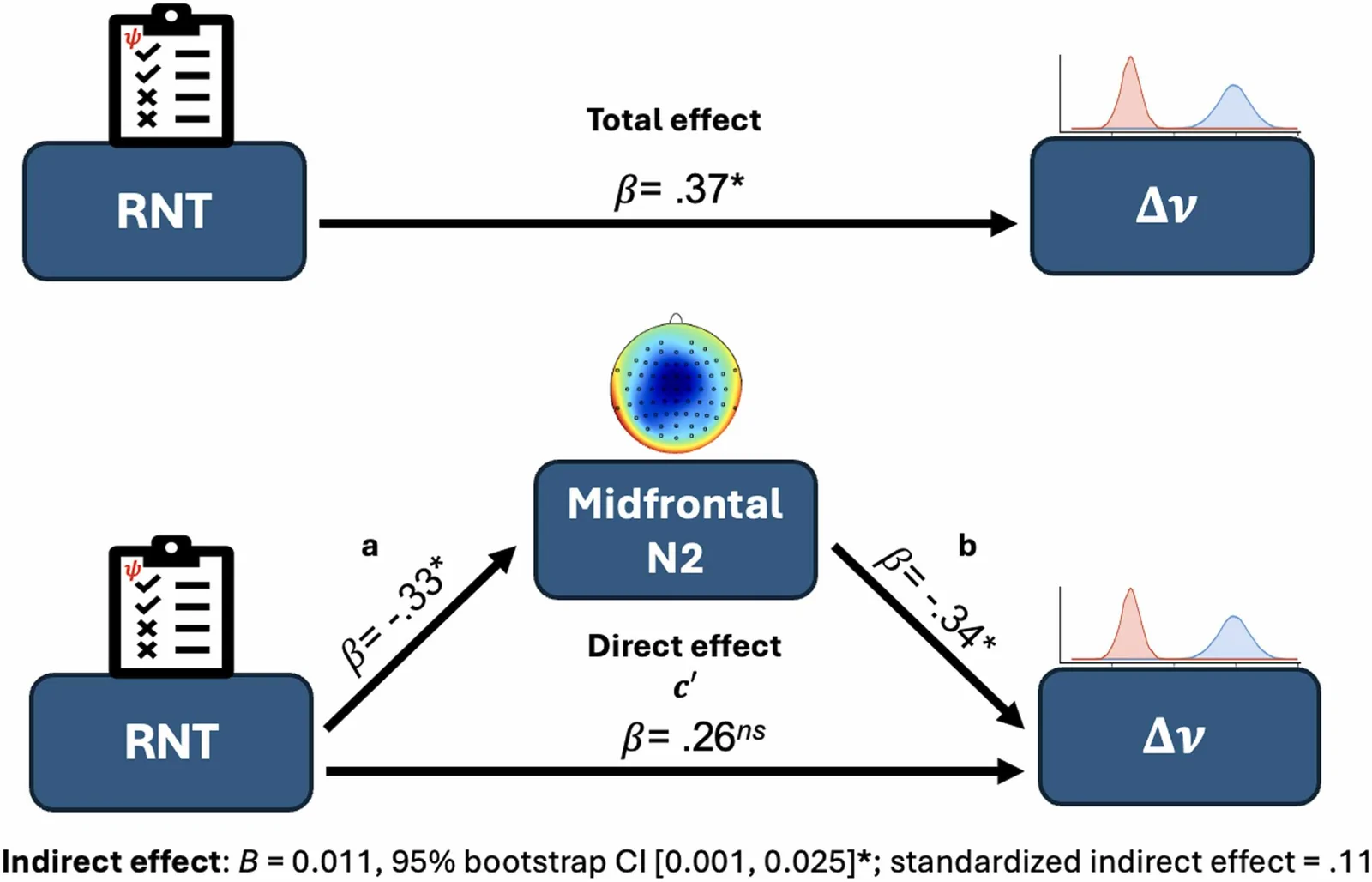
Midfrontal N2 mediates the relationship between RNT and drift-rate congruency effect. Path diagram depicts the total, direct, and indirect effect of RNT on the drift rate congruency effect (Δν) through midfrontal N2 conflict activity (incongruent – congruent). Path coefficients are standardized estimates (β); the indirect effect is reported as the unstandardized coefficient (*B*) with its 95% bootstrap confidence interval and standardized indirect effect. * = 95% bootstrap confidence interval excluded zero, ns = 95% bootstrap confidence interval included zero.

## 4 Discussion

The current study examined whether RNT is associated with conflict processing using a multimethod approach combining EEG and drift diffusion modeling. At the behavioral and EEG level, participants showed the robust flanker congruency effect in accuracy, RT, and the midfrontal N2 ERP, confirming that the task successfully engaged conflict processing. However, RNT was unrelated to the behavioral measures of accuracy and RT, indicating that these metrics may lack the sensitivity to detect individual differences in conflict processing associated with RNT. In contrast, analyses indicated that RNT was associated with larger congruency-related differences in drift rate, suggesting that higher RNT is associated with a wider evidence accumulation gap between low and high conflict situations. At the neural level, RNT was associated with stronger midfrontal N2 conflict activity (more negative voltage), and N2 amplitude was further associated with drift rate congruency effects. The relationship between RNT and drift rate congruency effect was supported by an exploratory mediation analysis that showed the midfrontal N2 mediated the association between RNT and the drift rate congruency effect, providing a candidate neural mechanism linking RNT to conflict-related decision dynamics. These patterns were not observed for individual differences in generalized anxiety symptoms or trait neuroticism, although a hierarchical regression analysis indicated a reduced effect of RNT after accounting for generalized anxiety symptoms and neuroticism. Collectively, these findings suggest that RNT is associated with heightened neural conflict-related activity that further disrupts the computational mechanisms underlying decision-making under conflict.

The current findings have important implications for understanding the neurocognitive mechanisms underlying RNT. First, RNT was unrelated to traditional flanker congruency effects on mean accuracy and RT, but was associated with the drift-rate congruency effect, suggesting that standard behavioral metrics may not be sensitive to detect subtle but important RNT-related differences in conflict processing [45]. Second, the influence of RNT on drift rate, where individuals with greater RNT showed larger congruency-related differences, may reflect a tendency to overprocess ambiguous or conflicting information relative to unambiguous information [46]. Notably, the drift rate effect may point to altered susceptibility to interference during evidence accumulation, such that individuals with higher RNT show a larger reduction in the efficiency of information processing when competing response information is present. This aligns with observations that individuals, prone to worry and rumination, core features of RNT, often function relatively well in predictable, low-demand situations but exhibit marked cognitive inefficiency when confronted with uncertainty, dual-task demands, or emotionally salient conflict [16,[47], [48], [49]]. Evidence accumulation models, such as the DDM, offer a computational framework to characterize these impairments by quantifying latent decision processes that are sensitive to cognitive control demands, and have been increasingly applied to internalizing disorders and repetitive negative thinking [50], revealing that individuals prone to rumination and worry often show inefficient or more variable evidence accumulation under conflict [51,52]. The present findings extend this literature by linking RNT-related differences in evidence accumulation to heightened neural conflict signaling. The results support the hypothesis that worry and rumination may, in part, arise from or be exacerbated by exaggerated conflict detection and inefficient decision making at both the neural and computational levels [14].

The mediation model revealed that individual differences in RNT were associated with drift-rate congruency effects via midfrontal N2 conflict amplitude, providing a candidate neural mechanistic account of how RNT affects conflict-driven cognitive control. Specifically, individuals with higher RNT exhibited higher negative conflict-related N2 amplitudes, a well-established neural marker of enhanced conflict detection [11,53], reflecting anterior cingulate activity [39,54]. Converging neuroimaging evidence links RNT to heightened ACC reactivity during cognitive control and conflict processing [55], in addition to altered functional connectivity between ACC/salience network and prefrontal regions [56,57], suggesting increased conflict sensitivity alongside disrupted cognitive engagement. Critically, the present findings extend this work by demonstrating that this amplified midfrontal conflict-related signal is associated with greater divergence in evidence accumulation under conflict, linking ACC-mediated conflict processing to downstream alterations in latent decision dynamics [23,58]. This view is consistent with integrative neurocomputational frameworks suggesting that frontal control signals bias or gate incoming evidence to support task-relevant choices when conflict is high [9,10,59]. Together, these findings suggest that RNT is characterized by heightened midfrontal sensitivity to conflict-related information with downstream consequences for the dynamic regulation of cognitive control under high versus low conflict demands [14,16].

These findings may also be relevant to dimensional models of internalizing disorders. RNT is a transdiagnostic process implicated across traditional diagnostic categories, including anxiety and depressive disorders, with elevated RNT associated with psychological distress across diagnostic categories [5,60]. RNT occurs across the general population but may become increasingly maladaptive when persistent and coupled with heightened emotion dysregulation, contributing to greater symptom burden and poorer treatment response [4]. Consistent with this dimensional approach, the present study recruited a community sample spanning a range of RNT and internalizing symptoms rather than selecting participants according to categorical psychiatric diagnoses. Notably, the mean GAD-7 score was 10.51, corresponding to moderate symptom severity [61], indicating meaningful internalizing symptom burden despite the absence of diagnostic assessment. Examining neural and computational correlates of RNT across this continuum is broadly consistent with dimensional frameworks such as the Research Domain Criteria (RDoC) and Hierarchical Taxonomy of Psychopathology (HiTOP), that emphasize mechanisms operating across traditional diagnostic categories and levels of symptom severity [62,63]. Within this dimensional framework, the observed associations with conflict processing were also more evident for RNT than for generalized anxiety symptoms or neuroticism. This may reflect RNT more proximally capturing perseverative cognitive processes implicated in conflict monitoring than broader measures of anxiety symptoms or negative emotionality [7]. However, the RNT effects were reduced after accounting for anxiety and neuroticism, limiting conclusions regarding RNT specificity.

From a clinical perspective, the current results have important implications for understanding the cognitive control deficits in individuals prone to RNT. First, these findings highlight limitations of standard behavioral measures for detecting RNT-related dysfunction and suggest that computational and neural markers of conflict processing may provide more sensitive assays for phenotyping and treatment predictions [64]. Second, these findings highlight potential treatment targets. Interventions aimed at improving cognitive control, such as adaptive working memory and interference control training may be particularly effective in reducing exaggerated responses to conflict, modifying latent decision-making markers, and reducing RNT [[65], [66], [67]]. More broadly, computational markers may provide a useful framework for predicting treatment response [68,69] and for evaluating whether interventions targeting cognitive control normalize underlying decision-making processes. Leveraging insights from computational models to inform and adapt behavioral therapies may also be useful [70].

There are limitations to our study that warrant comment. First, although the mediation model provides a mechanistic framework linking RNT, conflict-related midfrontal neural activity, and evidence accumulation, the cross-sectional design precludes making causal statements, and alternative explanations or unmeasured third-variable influence cannot be ruled out. Longitudinal or experimental studies targeting conflict-related processes will be important for establishing causal relationships. Second, the present findings are based on a specific implementation of the DDM, and alternative modeling approaches such as jointly modeling EEG and behavioral data [71], extensions of the DDM that further parameterize attention [72], or models that account for competing evidence accumulators [73] may provide important insights. Third, the sample size is relatively modest. While comparable to previous EEG-DDM studies [23,26], replication in larger samples and in clinical populations will be important for establishing the generalizability, robustness, and clinical utility of these findings.

In summary, the present study adds to the growing literature on the neurocognitive mechanisms underlying RNT. These findings provide novel evidence that individual differences in RNT are associated with conflict-related divergence in latent decision-making processes, mediated through heightened conflict-related neural activity. By linking midfrontal conflict activity to alterations in evidence accumulation, the current results offer a mechanistic framework for understanding how RNT impacts cognitive control. Elucidating these mechanisms may provide important insight into the vulnerability and maintenance of neuropsychiatric disorders characterized by maladaptive negative thinking.

## Ethics declaration

Written informed consent to take part in the study and to publish the article has been obtained from all participants or their legal representatives. The privacy rights of participants have been observed.

This study was performed in compliance with relevant laws, regulatory frameworks and guidelines where the research took place. This study was approved by the University of California, San Diego. (Approval No. 803050)

## Declaration of Generative AI and AI-assisted technologies in the writing process

Generative AI tools, including ChatGPT and Claude, were used in a limited capacity to assist with portions of the R code used for data analysis and visualization. No study data files or participant-level data were uploaded to or entered into these platforms. All AI-assisted code was reviewed, verified, and edited by the authors as needed. The authors take full responsibility for the integrity of the analyses and the content of the published article.

## Declaration of Competing Interest

The authors have no financial or personal relationships to disclose that influence or bias the work presented in this manuscript.

## Data Availability

Data and code will be made available upon reasonable request and subject to approved institutional data-use agreements.
