# Supplementary material for: Repetitive negative thinking is associated with conflict-related neural activity that modulates evidence accumulation
Source: J Mood Anxiety Disord. 2026 Sep 20;16:100206. doi: 10.1016/j.xjmad.2026.100206 (PMC13633890; doi:10.1016/j.xjmad.2026.100206)
Supplement: Supplementary file 1 [file mmc1.docx]

**SUPPLEMENTARY MATERIALS**

**For**

**Repetitive Negative Thinking is Associated with Conflict-Related Neural Activity that Modulates Evidence Accumulation**

Linisa Williams^1^; Jessica Bomyea^2,3^; Jonathon R. Howlett^2,3^; Katia M. Harlé^2,3^; Daniel M. Stout^2,3^*

^1^Department of Neurosciences, University of California San Diego

^2^Department of Psychiatry, University of California San Diego

^3^Center of Excellence for Stress and Mental Health, VA San Diego Healthcare System

***Corresponding Author**

**Address correspondence to:**

Dr. Daniel M. Stout (dastout@health.ucsd.edu)

Center of Excellence for Stress and Mental Health

VA San Diego Healthcare System

3350 La Jolla Village Drive

San Diego, CA 92161

| **Supplemental Table 1. Summary of hierarchical DDM parameterization across models** | | | | | |
| --- | --- | --- | --- | --- | --- |
| **Model** | **Drift rate (**$\boldsymbol{\nu}$**)** | **Boundary separation (*bs*)** | **Non-decision time (*ndt*)** | **Group-level parameters** | **Subject-level parameters** |
| Model 1 | Congruency-  specific (2) | Intercept only (1) | Intercept only (1) | 4 | Random slopes for $\nu$ (2); random intercepts for *bs*, *ndt* (2) |
| Model 2 | Congruency-  specific (2) | Congruency-  specific (2) | Intercept only (1) | 5 | Random slopes for $\nu$ and *bs* (4); random intercepts for *ndt* (1) |
| Model 3 | Congruency-  specific (2) | Congruency-  specific (2) | Congruency-specific (2) | 6 | Random slopes for $\nu$, *bs*, ndt (6) |
| ***Note***. Values in parentheses reflect count. Group-level parameters refer to condition-specific coefficients or intercepts. Subject-level parameters refer to standard deviations of random effects. Correlations among random effects were not estimated. | | | | | |

| **Supplemental Table 2**. MCMC convergence statistics for hierarchical DDM models | | | | | |
| --- | --- | --- | --- | --- | --- |
| **Model** | **Parameter** | **Estimate [95% CrI]** | $\hat{\mathbf{R}}$ | **Bulk ESS** | **Tail ESS** |
| **Model 1** | $\nu$(Congruent) | 3.72 [3.28-4.14] | 1.01 | 567 | 1102 |
|  | $\nu$(Incongruent) | 2.37 [2.06-2.68] | 1.01 | 672 | 1236 |
|  | *bs* | 1.23 [1.14-1.35] | 1.01 | 720 | 1521 |
|  | *ndt* | 0.24 [0.23-0.24] | 1.00 | 1129 | 1965 |
| **Model 2** | $\nu$(Congruent) | 3.87 [3.39-4.31] | 1.00 | 888 | 1758 |
|  | $\nu$(Incongruent) | 2.33 [1.98-2.67] | 1.00 | 834 | 1659 |
|  | *bs*(Congruent) | 1.30 [1.19-1.40] | 1.00 | 1066 | 2372 |
|  | *bs*(Incongruent) | 1.22 [1.13-1.34] | 1.01 | 795 | 1751 |
|  | *ndt* | 0.24 [0.23-0.24] | 1.00 | 1601 | 2687 |
| **Model 3*** | $\nu$(Congruent) | 3.96 [3.42-4.46] | 1.00 | 932 | 1827 |
|  | $\nu$(Incongruent) | 2.30 [1.98-2.63] | 1.01 | 817 | 1659 |
|  | *bs*(Congruent) | 1.39 [1.26-1.54] | 1.00 | 1060 | 2103 |
|  | *bs*(Incongruent) | 1.17 [1.09-1.27] | 1.00 | 1115 | 1946 |
|  | *ndt*(Congruent) | 0.23 [0.23-0.24] | 1.00 | 2611 | 4556 |
|  | *ndt*(Incongruent) | 0.24 [0.24-0.25] | 1.01 | 1199 | 2417 |
| ***Note.*** Estimates reflect posterior means and 95% credible intervals (CrI). Boundary separation (*bs*) and non-decision time (*ndt*) were estimated on the log scale and exponentiated for interpretability. Bulk ESS and Tail ESS quantify effective sample size in the center and tails of the posterior distribution. *Best Fitting model (see main report for details). | | | | | |

**Supplemental Figure 1. Posterior predictive check of RT from Model 3.** Grouped density overlays compare observed (*y*) reaction time (RT) distributions with posterior predictive distributions simulated (*y*_rep_; n draws = 200) from the model separately for congruent and incongruent trials.

**Supplemental Figure 2. Trace plots for primary fixed-effects parameters from Model 3.** Boundary separation and non-decision time are shown on the model-estimated log scale.

**Model 3 sensitivity analysis**

Given the unexpected finding of greater boundary separation for congruent than incongruent trials, we conducted a post hoc sensitivity analysis extending Model 3 to additionally estimate across-trial variability in start point (*s_z_),* while retaining congruency-specific drift rate, boundary separation, and non-decision time and fixing mean starting-point bias at 0.5. Starting point variability was substantial (*s_z_* = 0.43), but the boundary-separation congruency effect was only minimally reduced ($\Delta log(bs)$=0.14, 95% CrI [0.005, 0.272]). Thus, allowing starting-point variability did not account for the unexpected boundary separation congruency effect, and the effect could not be clearly interpreted as reflecting differences in response caution between congruent and incongruent trials. Given this uncertain interpretation, boundary separation was not examined in relation to RNT or midfrontal N2 activity.

| **Supplemental Table 3.** Sensitivity Model including variability in start-point | | | | |
| --- | --- | --- | --- | --- |
| **Parameter** | **Estimate [95% CrI]** | $\hat{\mathbf{R}}$ | **Bulk ESS** | **Tail ESS** |
| $\nu$(Congruent) | 4.49 [3.79-5.05] | 1.01 | 519 | 1,155 |
| $\nu$(Incongruent) | 2.70 [2.31-3.07] | 1.01 | 607 | 1,156 |
| *bs*(Congruent) | 1.47 [1.34-1.63] | 1.00 | 741 | 1,598 |
| *bs*(Incongruent) | 1.28 [1.18-1.40] | 1.01 | 683 | 1,371 |
| $\boldsymbol{\Delta log(}$***bs) (Cong – Incong)*** | **0.14 [0.005-0.272]** |  |  |  |
| *ndt*(Congruent) | 240 [238-241] | 1.00 | 3,738 | 4,154 |
| *ndt*(Incongruent) | 144 [83-198] | 1.00 | 7,568 | 5,706 |
| *s_z_* | 0.43 [0.40-0.45] | 1.00 | 5,979 | 5,561 |
| ***Note*.** Estimates are posterior medians with 95% credible intervals (CrI). The sensitivity model extended Model 3 by additionally estimating across-trial variability in starting point (*s_z_*), while mean starting-point bias remained fixed at 0.5. Non-decision time is reported in milliseconds, Ř = Gelman-Rubin convergence statistic; Bulk ESS = bulk effective sample size; Tail ESS = tail effective sample size. | | | | |

**Supplemental Figure 3. Pearson *r* scatter plots depicting associations between PTQ, DDM parameters, and conflict N2.** *P*-values reported are uncorrected.
